# In silico evaluation of the interaction between ACE2 and SARS-CoV-2 Spike protein in a hyperglycemic environment

**DOI:** 10.1038/s41598-021-02297-w

**Published:** 2021-11-24

**Authors:** Giovanni Sartore, Davide Bassani, Eugenio Ragazzi, Pietro Traldi, Annunziata Lapolla, Stefano Moro

**Affiliations:** 1 Department of Medicine (DIMED), University of Padova School of Medicine and Surgery, Via Giustiniani 2, 35128 Padua, Italy; 2 Department of Pharmaceutical and Pharmacological Sciences (DSF), Molecular Modeling Section (MMS), University of Padova School of Medicine and Surgery, Via Marzolo, 5, 35131 Padua, Italy; 3 Department of Pharmaceutical and Pharmacological Sciences (DSF), University of Padova School of Medicine and Surgery, Largo Meneghetti 2, 35131 Padua, Italy; 4 Nano-Inspired Biomedicine Lab, Fondazione Istituto di Ricerca Pediatrica Città della Speranza, Corso Stati Uniti 4, 35127 Padua, Italy

**Keywords:** Computational biology and bioinformatics, Endocrinology, Molecular medicine

## Abstract

The worse outcome of COVID-19 in people with diabetes mellitus could be related to the non-enzymatic glycation of human ACE2, leading to a more susceptible interaction with virus Spike protein. We aimed to evaluate, through a computational approach, the interaction between human ACE2 receptor and SARS-CoV-2 Spike protein under different conditions of hyperglycemic environment. A computational analysis was performed, based on the X-ray crystallographic structure of the Spike Receptor-Binding Domain (RBD)-ACE2 system. The possible scenarios of lysine aminoacid residues on surface transformed by glycation were considered: (1) on ACE2 receptor; (2) on Spike protein; (3) on both ACE2 receptor and Spike protein. In comparison to the native condition, the number of polar bonds (comprising both hydrogen bonds and salt bridges) in the poses considered are 10, 6, 6, and 4 for the states ACE2/Spike both native, ACE2 native/Spike glycated, ACE2 glycated/Spike native, ACE2/Spike both glycated, respectively. The analysis highlighted also how the number of non-polar contacts (in this case, van der Waals and aromatic interactions) significantly decreases when the lysine aminoacid residues undergo glycation. Following non-enzymatic glycation, the number of interactions between human ACE2 receptor and SARS-CoV-2 Spike protein is decreased in comparison to the unmodified model. The reduced affinity of the Spike protein for ACE2 receptor in case of non-enzymatic glycation may shift the virus to multiple alternative entry routes.

## Introduction

Type 2 diabetes mellitus has been considered a risk factor for acquiring the SARS-CoV-2 infection^1^. Increased morbidity in Type 2 diabetes mellitus has been documented since the initial spread of the pandemy and people with preexisting Type 2 diabetes mellitus have an increased need for medical intervention^2^. Meta-analyses and literature reviews have confirmed that patients with diabetes mellitus have higher risk of COVID-19 disease severity and mortality^3–6^. The reason for this phenomenon is still under debate^6–9^. Among the hypotheses available, one considers that people affected by diabetes mellitus have an increased risk of severe COVID-19 disease due to an imbalance between ACE1 and ACE2 activity^1^ which leads to pro-inflammatory responses, predisposing to the cytokine storm syndrome^1,10^. Increased ACE2 expression has been observed in patients with diabetes mellitus and COVID-19, as well as an increased pro-inflammatory profile^11^. It has been suggested that an upregulation of ACE2 receptors may favour viral entry into host cells, conditioning a higher viral load and poor prognosis; also, the loss of physiological function of ACE2 may enhance systemic adverse effects of the renin-angiotensin aldosterone system^12^. However, data on the mechanisms that drive the COVID-19 severity linked to ACE2 pathways in diabetes mellitus are still lacking. In addition, the excess of adipose tissue, typical of type-2 diabetes mellitus, is associated with increased macrophage and T-cell activation, together with increased proinflammatory cytokine production^13^.

Regarding COVID-19 in patients affected by diabetes mellitus, little attention seems to be paid to the mechanisms of SARS-CoV-2 interaction with access sites. In a previous paper, we hypothesized that a worse outcome of COVID-19 in people with diabetes mellitus could be related to the non-enzymatic glycation of human ACE2, which could trigger the activity of ACE2 to a more susceptible interaction with virus Spike protein^14^. Recent findings that about half of hospitalized patients with Type 2 diabetes mellitus present a myocardial damage have suggested a specific role played by glycated ACE2 receptor^15^. On the other side, “glycosilated” ACE2 receptor has been documented^16^ and glycosylation status has been considered as a possible determinant in SARS-CoV-2 infection susceptibility^17^. With the term “glycation”, we refer here to the modification of lysine caused by the Maillard reaction followed by the rearrangement of the Amadori products obtained^18,19^. Advanced Glycation End products (AGEs), which are produced by glycation of cellular molecules, including proteins, have been linked to increased COVID-19 risk factors^9^. Based on our hypothesis^14^, an upregulation of ACE2, due to its non-enzymatic glycation, together with a variation of the protein tertiary structure due to the aforementioned aminoacidic modifications, was suggested as a pathogenetic mechanism of SARS-CoV-2 negative outcome in diabetes mellitus.

The present work aimed to evaluate, through a computational approach, the interaction between human ACE2 receptor and SARS-CoV-2 Spike protein under different conditions of the hyperglycemic environment, which has been shown to influence the non-enzymatic glycation of the lysine residues of the aforementioned proteins. Looking deeply into the complexes deposited in the Protein Data Bank (PDB), such as 6LZG or 6M0J, considering the 34 lysine residues present in the extracellular portion of the ACE2 enzyme, a conformational change caused by non-enzymatic glycation could be possible. In addition, it is important to remember that also Spike protein has several lysine residues present on its surface, and a change in its tertiary structure due to glycation should also be considered.

## Methods

To assess if the glycation of the lysine aminoacid residues of ACE2 receptor or Spike protein Receptor Binding Domain (RBD) could affect their interaction, computational analysis was performed. First of all, the X-Ray crystallographic structure of the Spike RBD-ACE2 system was downloaded from Protein Data Bank (PDB: 6M0J, X-ray resolution: 2.45 Å, DOI: 10.2210/pdb6M0J/pdb)^20^. This system was properly prepared with Molecular Operating Environment (MOE) Structure Preparation Tool. The missing loops were rebuilt exploiting the MOE loop builder application. This program is able to create small missing parts of protein structures based on their sequence. Each loop created is then subjected to a multi-stage energy optimization, firstly aiming to remove the clashes in its structure and then to minimize it in the overall system, which is kept fixed with the exeption of the 3 nearest residues in both sides of the loop examined. The orientation of residues with alternative conformational states was chosen based on the occupancy. The hydrogen atoms were added with the MOE Protonate 3D tool, and the same program was used to assess the most probable protonation state at pH 7.4. The protonation and flip states are taken from a database of states which is built in MOE. Among the states present in this database, the Protonate 3D program selects the most probable one for each aminoacid evaluating specifically its own environment. The protonation predictions exploit a Generalized Born implicit solvation model, which is able to take into account the long-range interactions and the solvation effects (for these calculations, in this experiment, the salt concentration parameter was set at 0.15 mol/L). The added hydrogen atoms were minimized using AMBER10:EHT^21^ force field implemented in MOE^22^.

It is important to underline that, in the present study, we considered the system without its glycosylated chains since glycan chains are very difficult to reproduce and are out of our main aim. A discussion of the role of glycans is reported in Supplementary Material 1.

To effectively compare the interactivity between the two protein interfaces in different glycation states, we decided to divide the computational study into three different parts. Firstly, we considered ACE2 receptor with lysine aminoacid residues transformed by glycation, then Spike protein with lysine aminoacid residues transformed by glycation, and finally we analyzed the situation with all lysines of the system subjected to glycation.

The analysis of each system was carried out both by visual inspection and by the intermolecular interactions count, exploiting the “GetContacts” tool (https://getcontacts.github.io/).

**Figure 1:**
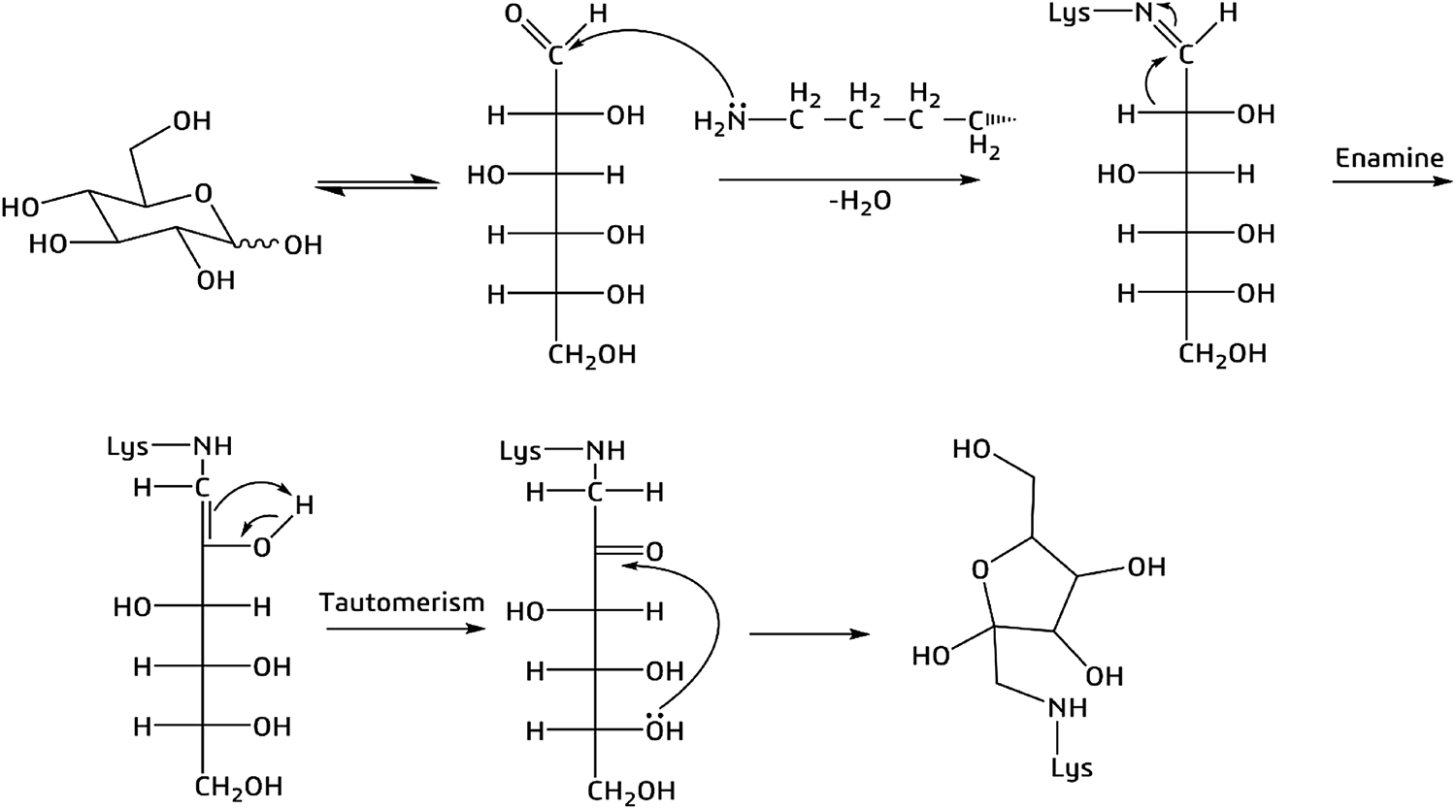
Mechanism of non-enzymatic glycation of a lysine amino acid residue by d-glucose. The final product is the cyclic amino sugar that we considered for the study.

As “glycation” we refer to the modification of lysine due to the Maillard reaction followed by the rearrangement of the Amadori products obtained^18,19^, and the derivative that we consider is the cyclic amino sugar, as represented in Fig. 1.

Both proteins of interest, SARS-CoV-2 Spike protein, and human ACE2 receptor, have lysine aminoacid residues on their surface, so all these sites were considered for glycation. In order to obtain images of easier interpretation, even if we considered all lysine aminoacid residues of the system, here we just report the results for the ones involved in the interaction.

To effectively analyze how interactivity changes with respect to glycation, we used the MOE protein–protein docking tool. The ACE2 protein was treated as the receptor and the RBD of Spike as the ligand. The ACE2 residues considered as the “binding site” were chosen according to Deganutti et al.^23^, to reproduce a proper binding situation between the two entities. For each system, 100 docking poses were generated, ranked, and visually inspected. The pose which could better reproduce the situation observable in 6M0J was selected and used for further analysis.

In order to obtain the glycated lysine residues for the study, the modified side chain was manually build using the Molecular Operating Environment “Builder” tool. The glycated residues obtained were then selectively minimized with the AMBER10:EHT^21^ force field implemented in MOE.

## Results

**Figure 2:**
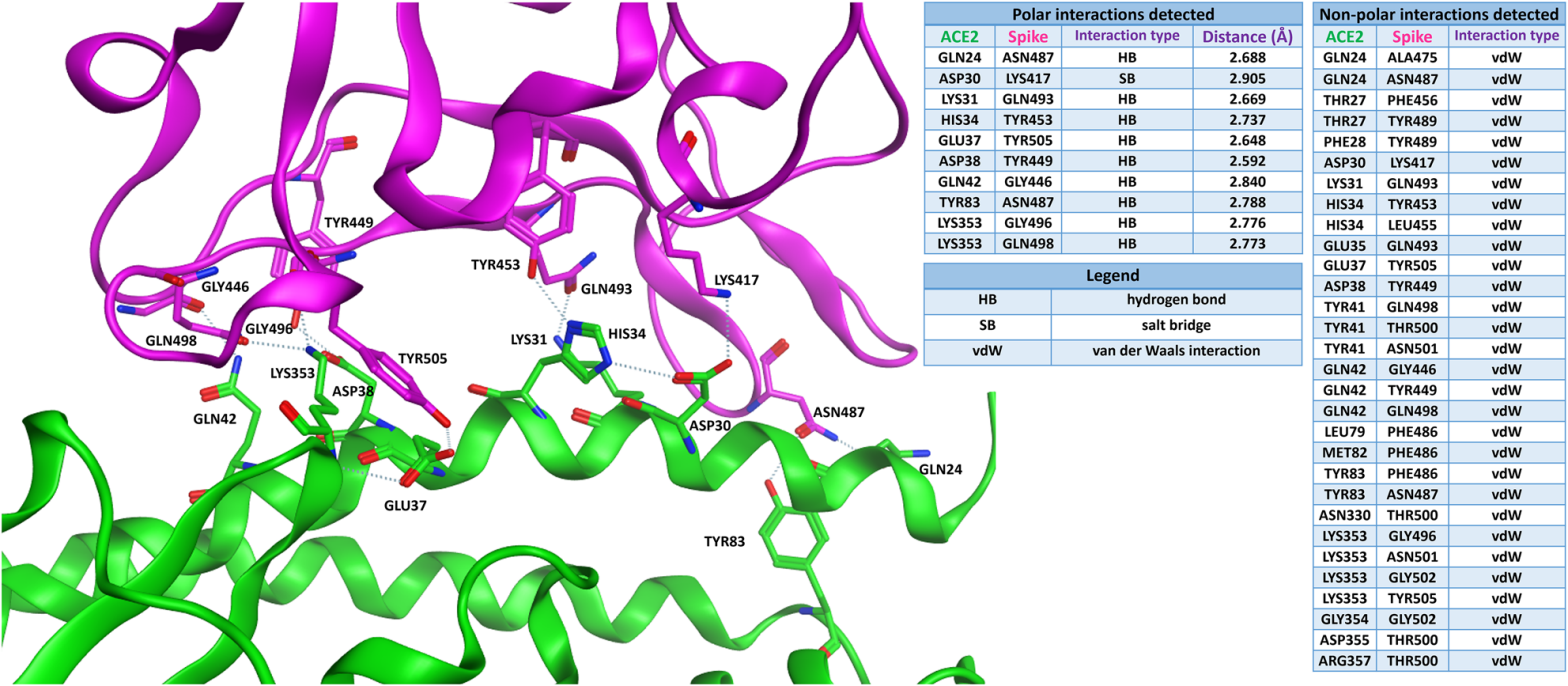
The typical situation of viral Spike protein RBD (PDB: 6M0J, violet-colored) binding to ACE2 receptor (green). The tables on the right report the polar and the non-polar interactions in which the residues on the interface are involved. For the polar interactions, also the distance between the interacting atoms (measured in angstroms) is reported in the table. To get a better visual representation of the contacts, just the residues engaged in the polar interactions are labelled in the 3D image on the left, while all the non-polar interactions are omitted.

Figure 2 shows the well-known interaction of Spike protein RBD (violet) with the native ACE2 receptor (green). The contacts established between the two proteins are reported in two different tables, one for polar interactions (namely, hydrogen bonds and salt bridges) and one for non-polar relationships (mainly van der Waals interactions). To get a better visual effect, just the residues involved in the polar interactions are labelled in the 3D image reported, together with the dashed lines indicating the polar contacts themselves. For the same reason, in the table regarding the non-polar relationships, just the aminoacid couples which interact between the proteins are indicated.

**Figure 3:**
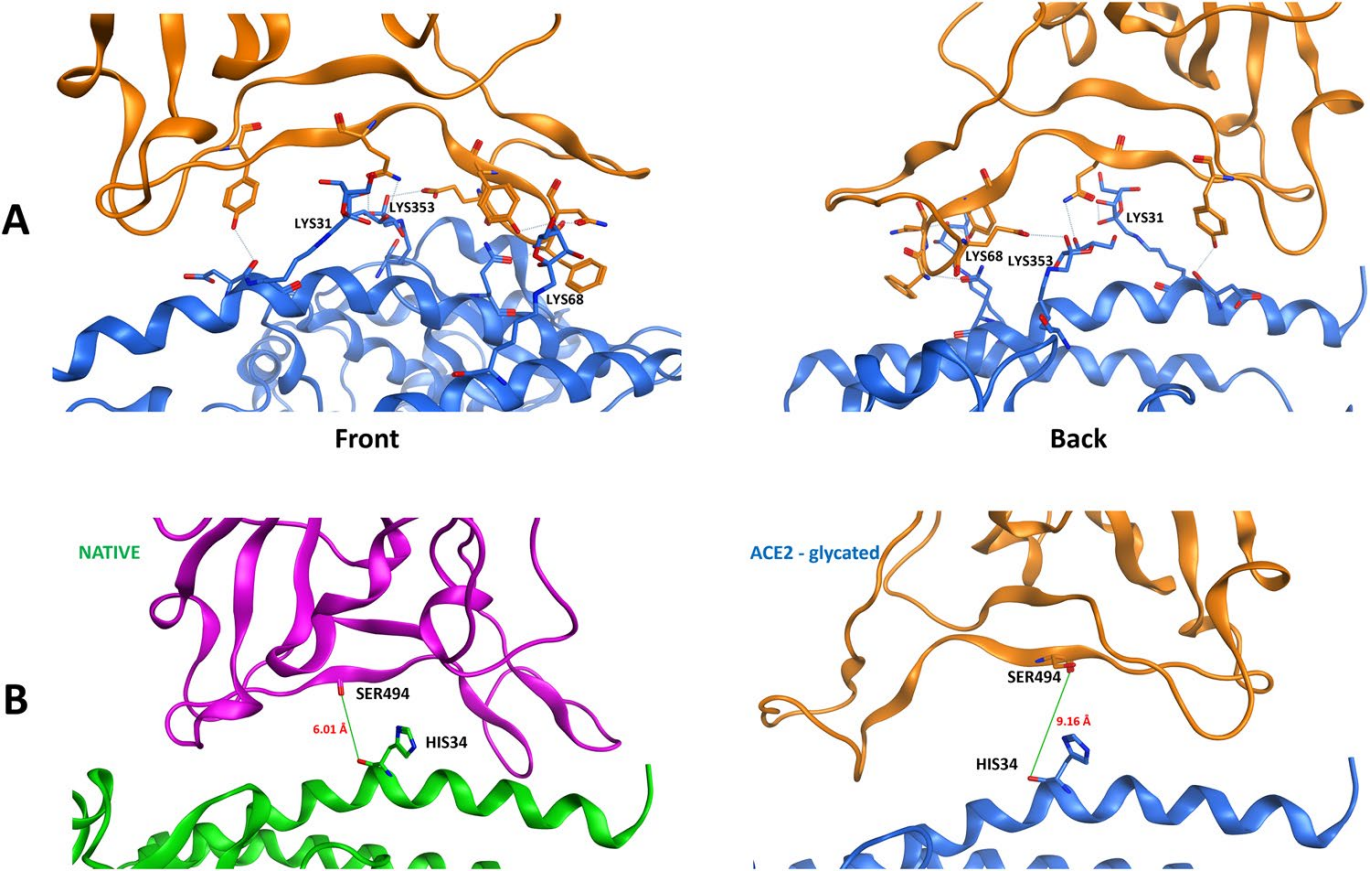
(**A**) Front and back highlight of the viral Spike protein (orange) binding to glycated ACE2 receptor (blue). The lysine amino acid residues subjected to glycation on ACE2 receptor are labelled in the image. (**B**) Difference in the distances between the first two cases considered. On the left: the classical interaction in PDB 6M0J, in which the distance between the carbonyl oxygen atoms of ACE2 receptor (green) HIS34 and Spike protein RBD (violet) SER494 is 6.01 Å. On the right: the interaction between glycated ACE2 receptor (blue) and native Spike protein RBD (orange). In this case, the distance between the carbonyl oxygen of ACE2 receptor (blue) HIS34 and Spike protein RBD (orange) SER494 is 9.16 Å.

The possible occurrence of glycation on ACE2 receptor (Fig. 3A) as suggested for diabetes mellitus condition, still allows a relevant binding of Spike protein RBD (orange) to the modified ACE2 (blue) site. Spatial differences in the binding, represented by the distance between the carbonyl oxygen atoms of Spike RBD SER494 and ACE2 HIS34, are shown in Fig. 3B.

**Figure 4:**
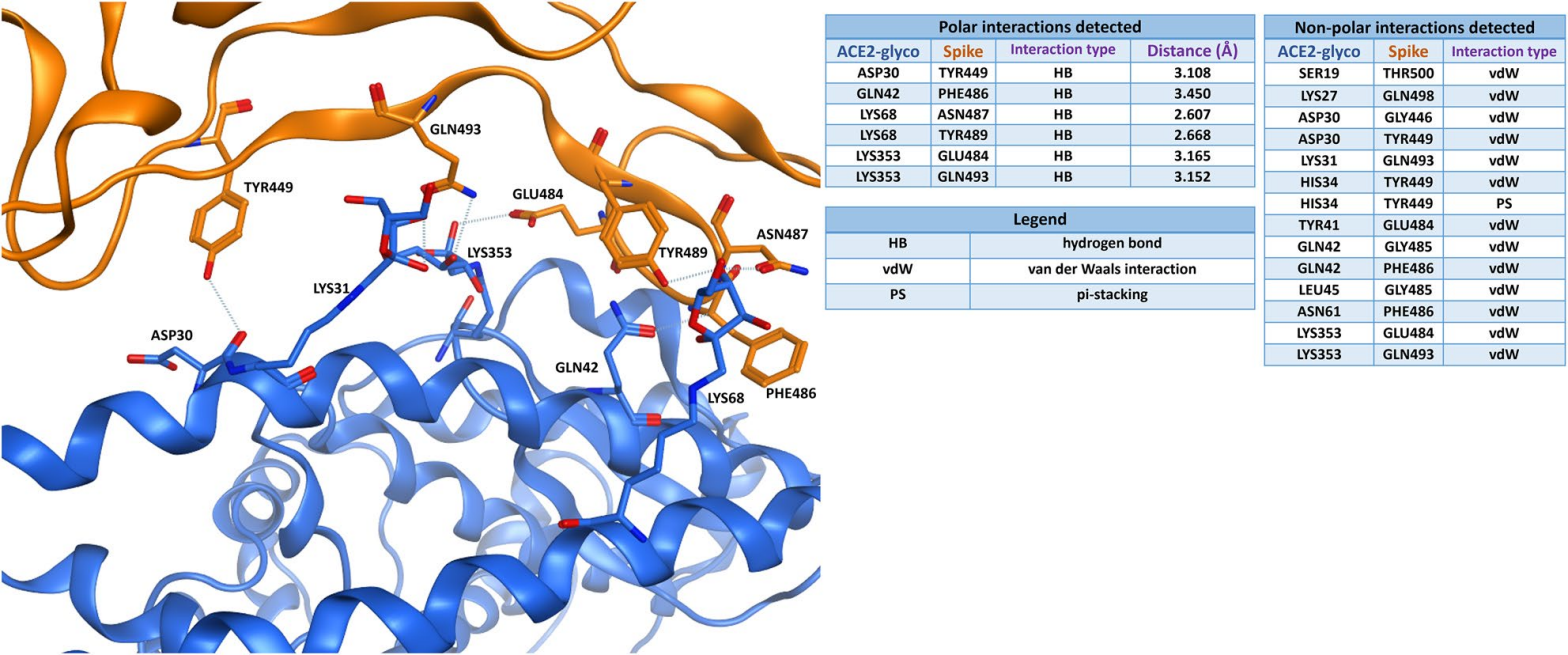
Viral Spike protein RBD (PDB: 6M0J, orange-colored) is binding to glycated ACE2 receptor (blue). The tables on the right report the polar and the non-polar interactions in which the residues on the interface are involved. For the polar interactions, also the distance between the interacting atoms (measured in angstroms) is reported in the table. To get a better visual representation of the contacts, just the residues engaged in the polar interactions are labelled in the 3D image on the left, while all the non-polar interactions are omitted.

Figure 4 presents the intermolecular interaction analysis of glycated ACE2 (blue) interaction with the native viral Spike RBD (not glycated, orange). As shown in the table, both the polar and the non-polar contacts between the proteins are decreased in comparison to the unmodified model of Fig. 2. Moreover, the distances between the atoms involved in the polar bonds tend to increase, and the only salt bridge establishing the interaction is lost.

**Figure 5:**
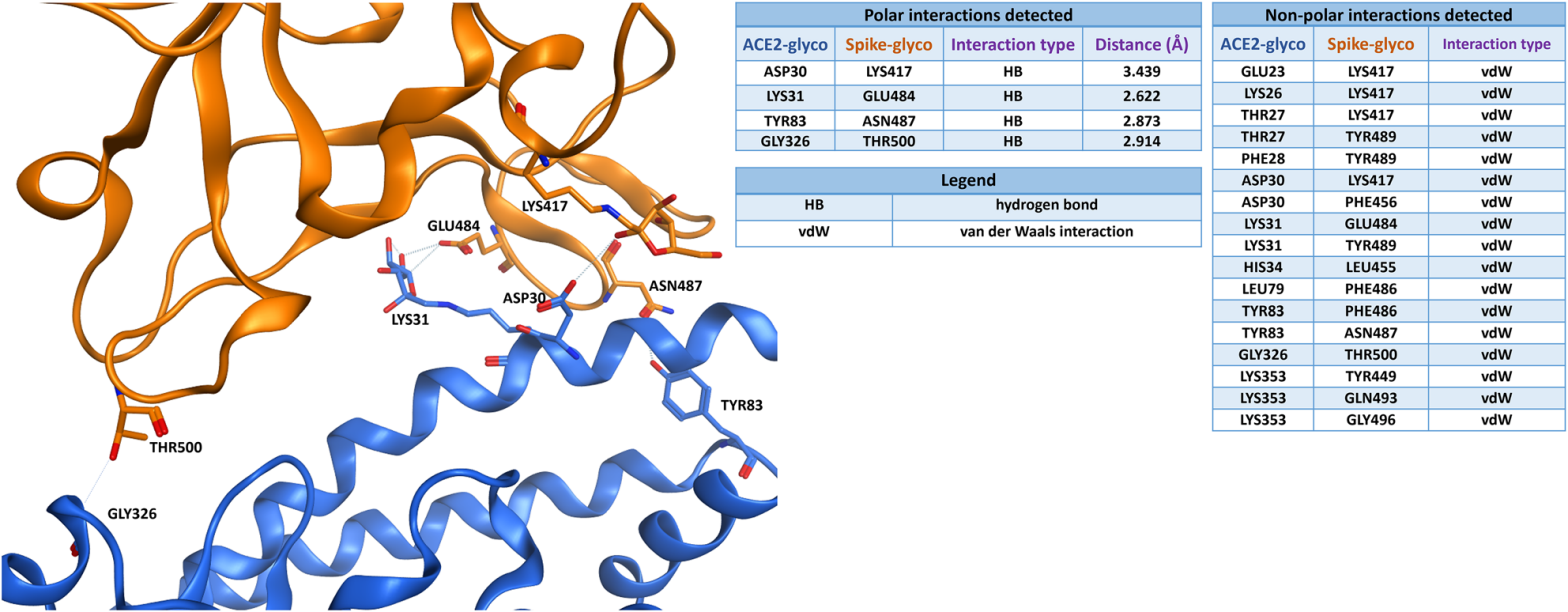
Glycated viral Spike protein RBD (orange) binding to glycated ACE2 receptor (blue). The tables on the right report the polar and the non-polar interactions in which the residues on the interface are involved. For the polar interactions, also the distance between the interacting atoms (measured in angstroms) is reported in the table. To get a better visual representation of the contacts, just the residues engaged in the polar interactions are labelled in the 3D image on the left, while all the non-polar interactions are omitted.

In the hypothesis that glycation may involve also Spike protein, a further evaluation was done. As shown in Fig. 5, the interaction analysis of glycated ACE2 receptor (blue) interaction with the glycated viral Spike RBD (orange) indicates a further decrease in the number of polar interactions involved, while the number of non-polar contacts is does not change significantly.

Further details of the molecular interactions in the different hypotheses of glycation, including the interaction of glycated Spike protein with native ACE2 (Figure S2.1), are reported in Supplementary material 2.

## Discussion

As a result of our in silico study, it appears that the glycation of the lysine aminoacid residues present both in Spike and ACE2 proteins lead to a loss of interaction between them, as is depictable with the analysis of the number of both polar and non-polar contacts. Considering all the possible scenarios of glycation of ACE2 receptor and/or Spike protein, it derives that the number of polar bonds in the poses considered are 10, 6, 6, and 4 for the states ACE2/Spike both native, ACE2 native/Spike glycated, ACE2 glycated/Spike native, ACE2/Spike both glycated, respectively. The number of non-polar contacts shows a very important decrease if the native model (in which 30 different aminoacid pairs establish non-polar interactions between the proteins) is compared with anyone of the others, while it does not show huge changes among the variously glycated models. Indeed, 14 aminoacid pairs which stabilize non-polar interactions can be depicted for the ACE2 glycated/Spike native system, 19 for the ACE2 native/Spike glycated model and 17 in the case in which both ACE2 and Spike proteins have lysines glycated. Looking deeply to the polar interactions, it is possible to evaluate an increase in the medium distance between the atoms involved in the contacts passing from the native model to the various glycated ones, suggesting a progressive interaction weakening. From data of the present analysis it could be concluded that non-enzymatic glycation of both ACE2 and Spike proteins exerts a detrimental effect on their interaction, independently on the fact that one or both proteins are involved in the glycation process. The analysis reported suggests that ACE2 glycation has a higher influence on the overall interactivity, mainly because the highest losses in non-polar interactions are depictable in the systems in which ACE2 has its lysine residues glycated.

Even if no experimental validation has been brought in the present work, further researches can benefit of the analysis here provided. Indeed, the results of the present study concur to enhance the knowledge about both the link between SARS-CoV-2 infection and diabetes mellitus and on SARS-CoV-2 Spike protein interaction with human ACE2^24^ at the molecular level.

Angiotensin-Converting Enzyme-2 (ACE2) is the most investigated site of SARS-CoV-2 access to the host cells^25,26^. SARS-CoV-2, compared to SARS-CoV, is characterized by a tighter bond to ACE2^20,25,27^, which can explain the high transmission rate of the SARS-CoV-2 virus^27^. The initial hypothesis that an increased risk of worse outcomes in patients with Type 2 diabetes mellitus is due to increased virus interaction with ACE2 sites following glycation^14^, seems however to be excluded, based on the present analysis.

Therefore, other possible explanations for disease severity of SARS-CoV-2 infection in diabetic patients may be provided. New additional evidence has suggested that alternative viral entry molecules are involved, both at viral and cellular level^28^. The interaction of SARS-CoV-2 with human cells is also dependent on Transmembrane Protease Serine-2 (TMPRSS2), a protein whose expression is regulated by androgen expression^29,30^, and that helps the virus enter into the host^26,28,31,32^. Moreover, it has been demonstrated that COVID-19 infection causes damage to the pancreatic tissue mediated by TMPRSS2^33^, further suggesting a pivotal role of the virus in the disease outcome in diabetic conditions. The reduced affinity of the Spike protein for the ACE2 receptor in the case of non-enzymatic glycation, as found in the present study, may shift the virus to alternative entry mechanisms, and TMPRSS2 could be one of these.

A common feature of viruses is their ability of developing new effective ways to penetrate into host cells when their main entry mechanism results to be slowed or impaired^34,35^. In this context, another described route of SARS-CoV-2 entry into cells, Neuropilin-1 (NRP1), could be considered^28,32,36–38^. NRP1 has been demonstrated as an additional SARS-CoV-2 infection mediator, in particular linked to the neurological aspects found in COVID 19^37^, but also because it is strictly involved in SARS-CoV-2 binding in lung tissue^39^ and in mediating diabetic nephropathy^40^. NRP1 has been found significantly up-regulated following SARS-CoV-2 infection^39^. The widespread presence of NRP1 in epithelial cells makes it a feasible entry pathway for the virus, and therefore in diabetes mellitus condition, when the glycated ACE2 receptor has lost at least part of its binding affinity for virus, NRP1 receptor could play a preferential, alternative, role of SARS-CoV-2 entry receptor.

Other proposed entry routes for SARS-CoV-2 include Dipeptidyl peptidase 4 (DPP4), also known as cluster of differentiation 26 (CD26), an ectopeptidase found in many tissues, such as lung and kidney, and involved in several physiological processes and diseases of the immune system^28,41^. A soluble form (sCD26) has been also described in blood, acting as protective factor against virus entry; sCD26 is reduced in diabetes mellitus, therefore conditioning an increased risk of infection in this population^41^. Different affinity for glycated ACE2 receptor may shift the virus entry through CD26. This mechanism could be involved in the reduced COVID-19 mortality observed with sitagliptin, a DDP-4 inhibitor drug used in Type 2 diabetes mellitus, by increasing the soluble form of DPP-4/sCD26^42^.

Another investigated receptor for SARS‐CoV‐2 infection is represented by the transmembrane glycoprotein CD147 (basigin 2), expressed in pathological tissues and in inflammation^28^. ACE2 and CD147 activities as entry pathways for SARS‐CoV‐2 are co-regulated, leading to downregulation following virus exposure^43^. Therefore, since CD147 expression is upregulated by high glucose concentrations and AGEs^44,45^, this alternative pathway may assume a pivotal role in conditioning virus binding to cells in diabetes mellitus.

Also glucose-regulated protein 78 (GRP78) and other receptors have been suggested as potential alternative receptors for SARS‐CoV‐2 entry into cells^46^. The existence of multiple pathways for virus binding can explain the altered susceptibility to COVID-19 in diabetes mellitus, after the present in silico analysis, suggesting decreased affinity for virus of glycated ACE2 receptor.

The present finding that glycation of ACE2 receptor reduces the affinity for Spike protein supports also the hypothesis that a downregulation of ACE2, observed after SARS‐CoV‐2 infection, leads to accumulation of angiotensin II and related metabolites^47^, conditioning the acute respiratory distress typical of COVID-19.

A role of Spike protein glycation, also possible in diabetes mellitus, and explored in the present study, might be hypothesized in conditioning the ability of SARS-CoV-2 to interact with the several possible access routes of the virus.

The scenario of possible interactions between viral Spike and host structures should also consider the fact that in diabetes mellitus the decomposition of glycated amino sugars, generated through the Maillard reaction, leads to intermediates of Advanced Glycation End (AGE) products, such as glyoxal and methylglyoxal^18,48^. These highly reactive carbonyl compounds, and others, formed also by degradation of glucose itself, can interact, even with greater reactivity, with proteins implicated in the virus access, and might further enhance the severity of SARS-CoV-2 infection in people affected by diabetes mellitus^9^. These considerations, supported by the present results, could help to explain why hyperglycemia worsens the prognosis of COVID-19, which has been also linked to the system of receptors for advanced glycation end products (RAGE)^49^. Moreover, a putative role of glycated hemoglobin, which is elevated in diabetic patients, has been suggested as an important factor for COVID-19 infection and mortality^50^. Evaluation of the possible reactive components of glucose metabolism may deserve further investigation.

An experimental evaluation of the hypothesis regarding glycated ACE2 and Spike interactions in diabetes mellitus goes beyond the purpose of the present work, being a strict computational approach, undertaken following observations of clinical and epidemiological data in the COVID-19 pandemy. The further hypotheses of a modified affinity of Spike protein in diabetes mellitus for alternative viral access mechanisms above discussed, which might become relevant also after Spike protein mutations, already documented^51,52^, have not been considered in the present study, and may deserve a future specific in silico evaluation followed by a possible experimental validation.

In conclusion, the present analysis supports the hypothesis that glycation, consequent to hyperglycemia in patients affected by diabetes mellitus, could have a role in the SARS-CoV-2 infection, possibly modulating other binding sites for SARS-CoV-2 access into the body.

## Supplementary Information

Supplementary Information 1.

Supplementary Information 2.

## Abbreviations

AGE: Advanced glycation end products
ACE1: Angiotensin-converting enzyme-1
ACE2: Angiotensin-converting enzyme-2
CD26: Cluster of differentiation 26
DPP4: Dipeptidyl peptidase 4
GRP78: Glucose-regulated protein 78
MOE: Molecular operating environment
NRP1: Neuropilin-1
PDB: Protein Data Bank
RAGE: Receptors for advanced glycation end products
RBD: Receptor binding domain
TMPRSS2: Transmembrane protease serine-2
