# Supplementary material for: In silico evaluation of the interaction between ACE2 and SARS-CoV-2 Spike protein in a hyperglycemic environment
Source: Sci Rep. 2021 Nov 24;11:22860. doi: 10.1038/s41598-021-02297-w (PMC8613179; doi:10.1038/s41598-021-02297-w)
Supplement: Supplementary file 1 — Supplementary Information 1. [file 41598_2021_2297_MOESM1_ESM.docx]

**Supplementary Material 1**

***In silico* evaluation of the interaction between ACE2 and SARS-CoV-2 Spike protein in a hyperglycemic environment**

Giovanni Sartore^1^, Davide Bassani^2^, Eugenio Ragazzi^3^, Pietro Traldi^4^, Annunziata Lapolla^1^, Stefano Moro^2^

^1^Department of Medicine (DIMED), University of Padova School of Medicine and Surgery, Via Giustiniani 2, 35128, Padova, Italy; G.S.: g.sartore@unipd.it; A.L.: annunziata.lapolla@unipd.it

^2^Department of Pharmaceutical and Pharmacological Sciences (DSF), Molecular Modeling Section (MMS), University of Padova School of Medicine and Surgery, Via Marzolo, 5, 35131, Padova, Italy; D.B.: davide.bassani.1@studenti.unipd.it; S.M.: stefano.moro@unipd.it

^3^Department of Pharmaceutical and Pharmacological Sciences (DSF), University of Padova School of Medicine and Surgery, Largo Meneghetti 2, 35131, Padova, Italy; E.R.: eugenio.ragazzi@unipd.it

^4^Nano-Inspired Biomedicine Lab, Fondazione Istituto di Ricerca Pediatrica Città della Speranza, Corso Stati Uniti 4, 35127 Padova, Italy; P.T.: p.traldi@irpcds.org

**The role of glycans in ACE2–Spike interaction**

The interaction of SARS-COV-2 Spike protein with ACE2 (angiotensin-converting enzyme 2) has been reported to represent one of the main mechanisms for the entrance of the virus in the host cell. Looking deeply into the structures of these two proteins (which interaction is represented in PDBs as 6LZG or 6M0J), it is important to remember that glycosylation is one of the main phases in the formation of both these. Indeed, it is reported that ACE2 has 6 diverse and heterogenic N-glycosylation sites in its extracellular region, while Spike protein is composed by 22 different canonical N-glycosylation sites [1]. As shown by recent works [1, 2], the interaction surface between Spike Receptor-Binding Domain (RBD) and ACE2 is highly influenced by the orientation of the carbohydrate chains nearby. One of the most important residues highlighted in this work is ACE2 Lys353 (Figure S1.1), located on the interface with Spike protein RBD. We depicted how its behavior changes in respect to non-enzymatic glycation, but how does it respond to changes in the glycosylation chains nearby is of more complex evaluation.


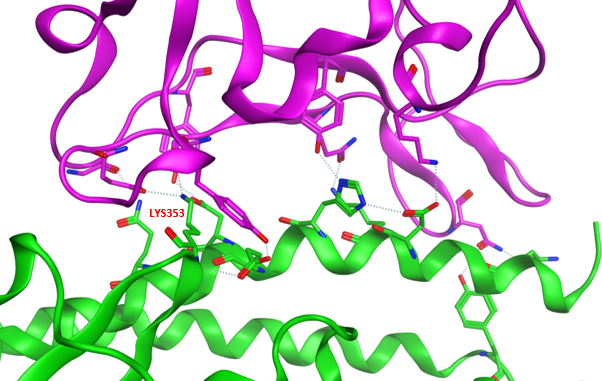


***Figure S1.1.*** *Viral Spike protein RBD (violet) bound to ACE2 receptor (green) in PDB 6M0J. LYS353, one of the most representative ACE2 lysine amino acids in the binding region, is labelled in red.*

This residue has also been highlighted in a recent study [3] as important in the interaction between Spike and ACE2.

If we look at the glycosylated structure of Spike (modeled from PBD: 6VSB) published in the work of Zhao et al. [1], we can have a representation of the volume occupied by the glycosylation chains. All these glycosylation sites are of course heterogeneous and make a great difference in both the shape and dimensions of the protein. When such highly glycosylated entities, such as Spike and ACE2, approach each other, both the composition and the dimensions of the glycosylated chains influence the binding event. Further experimental evaluation, supported by computational techniques, could depict the magnitude with which each change in the glycosilation influences the overall stability of the complex, but such analysis is of high complexity and is out of our main goal for this work.

**References**

[1] P. Zhao *et al.*, “Virus-Receptor Interactions of Glycosylated SARS-CoV-2 Spike and Human ACE2 Receptor,” *Cell Host Microbe*, vol. 28, no. 4, Oct. 2020, doi: 10.1016/j.chom.2020.08.004.

[2] L. Casalino *et al.*, “Beyond Shielding: The Roles of Glycans in the SARS-CoV-2 Spike Protein,” *ACS Cent. Sci.*, vol. 6, no. 10, Oct. 2020, doi: 10.1021/acscentsci.0c01056.

[3] R. Yan, Y. Zhang, Y. Li, L. Xia, Y. Guo, and Q. Zhou, “Structural basis for the recognition of SARS-CoV-2 by full-length human ACE2,” *Science (80-. ).*, vol. 367, no. 6485, Mar. 2020, doi: 10.1126/science.abb2762.
