# Supplementary material for: In silico evaluation of the interaction between ACE2 and SARS-CoV-2 Spike protein in a hyperglycemic environment
Source: Sci Rep. 2021 Nov 24;11:22860. doi: 10.1038/s41598-021-02297-w (PMC8613179; doi:10.1038/s41598-021-02297-w)
Supplement: Supplementary file 2 — Supplementary Information 2. [file 41598_2021_2297_MOESM2_ESM.docx]

**Supplementary Material 2**

***In silico* evaluation of the interaction between ACE2 and SARS-CoV-2 Spike protein in a hyperglycemic environment**

Giovanni Sartore^1^, Davide Bassani^2^, Eugenio Ragazzi^3^, Pietro Traldi^4^, Annunziata Lapolla^1^, Stefano Moro^2^

^1^Department of Medicine (DIMED), University of Padova School of Medicine and Surgery, Via Giustiniani 2, 35128, Padova, Italy; G.S.: g.sartore@unipd.it; A.L.: annunziata.lapolla@unipd.it

^2^Department of Pharmaceutical and Pharmacological Sciences (DSF), Molecular Modeling Section (MMS), University of Padova School of Medicine and Surgery, Via Marzolo, 5, 35131, Padova, Italy; D.B.: davide.bassani.1@studenti.unipd.it; S.M.: stefano.moro@unipd.it

^3^Department of Pharmaceutical and Pharmacological Sciences (DSF), University of Padova School of Medicine and Surgery, Largo Meneghetti 2, 35131, Padova, Italy; E.R.: eugenio.ragazzi@unipd.it

^4^Nano-Inspired Biomedicine Lab, Fondazione Istituto di Ricerca Pediatrica Città della Speranza, Corso Stati Uniti 4, 35127 Padova, Italy; P.T.: p.traldi@irpcds.org


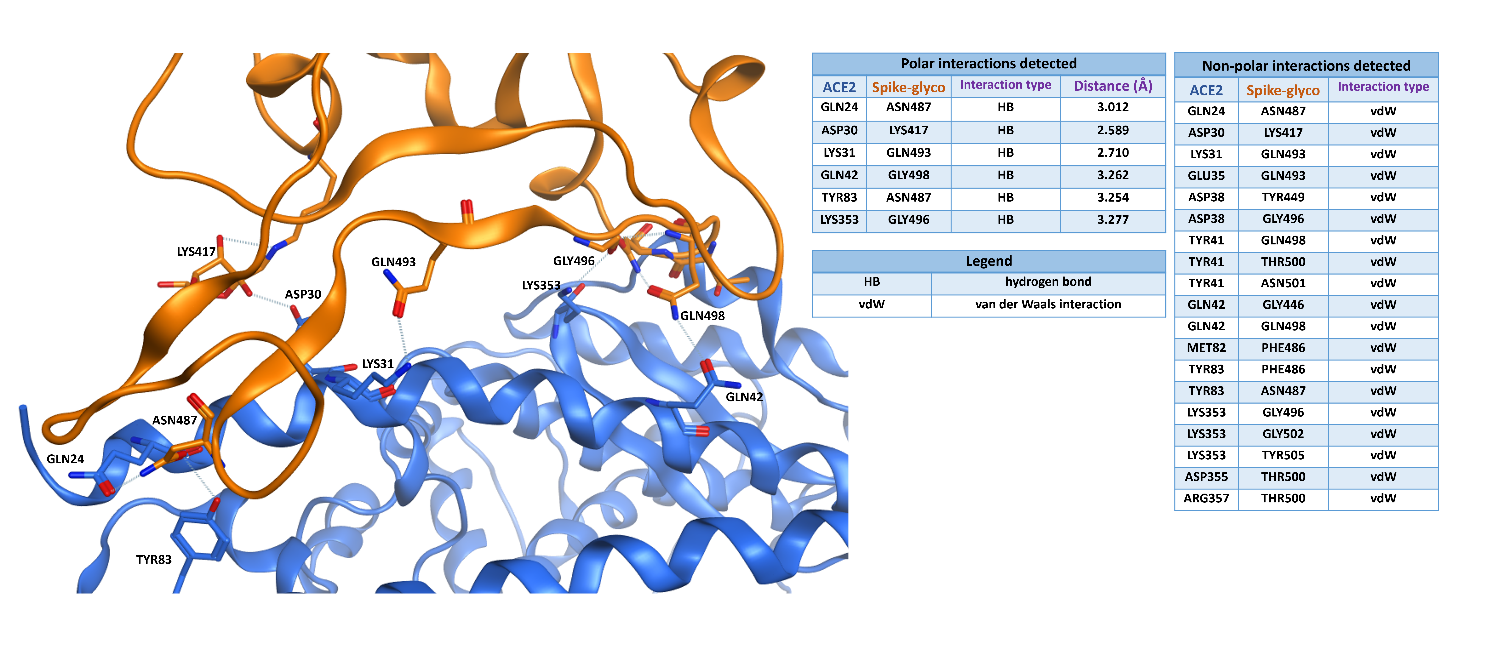
To complete the study, we also report in Figure S2.1 the results for the case in which the glycated form of Spike RBD (orange) is bound to a native ACE2 receptor (blue). In this case, even if we observe a reduction in the number of polar bonds in respect to the native model, we notice that this reduction is comparable with the case in which the native Spike RBD interacts with a glycated ACE2 receptor The number of non-polar interactions is higher than in the ACE2 glycated/Spike native case (19 contacts vs 14). This could suggest that glycation affects the interaction between the proteins in a stronger manner when involving the receptor ACE2 rather than Spike protein.

***Figure S2.1.*** *Glycated viral Spike protein RBD (orange) binding to native ACE2 receptor (blue). The tables on the right report the polar and the non-polar interactions in which the residues on the interface are involved. For the polar interactions, also the distance between the interacting atoms (measured in Angstroms) is reported in the table. To get a better visual representation of the contacts, just the residues engaged in the polar interactions are labelled in the 3D image on the left, while all the non-polar interactions are omitted.*
